# The Impact of *KRAS* Mutational Status on Long-Term Survival following Liver Resection for Hilar Cholangiocarcinoma

**DOI:** 10.3390/cancers14184370

**Published:** 2022-09-08

**Authors:** Francesco Ardito, Francesco Razionale, Andrea Campisi, Angela Carlino, Maria Vellone, Simone Vani, Luigi M. Larocca, Felice Giuliante

**Affiliations:** 1 Hepatobiliary Surgery Unit, Foundation Policlinico Universitario A. Gemelli, IRCCS, 00168 Rome, Italy; 2 Department of Translational Medicine and Surgery, Università Cattolica del Sacro Cuore, 00168 Rome, Italy; 3 Department of Pathology, Foundation Policlinico Universitario A. Gemelli, IRCCS, 00168 Rome, Italy

**Keywords:** *KRAS* mutational status, perihilar cholangiocarcinoma, Klatskin tumor, liver resection, overall survival, personalized medicine

## Abstract

**Simple Summary:**

The reported frequency of *KRAS* mutations and their prognostic impact in patients resected for hilar cholangiocarcinoma are controversial. The aim of this study was to evaluate the rate of *KRAS* mutations in a single-center homogeneous population resected for hilar cholangiocarcinoma and the impact on prognosis. The frequency of *KRAS* mutations was 22.2%. *KRAS* mutation was not related with pathologic characteristics of the tumor. Patients with a *KRAS* mutation presented zero 5-year OS that was significantly lower than that observed in patients with *KRAS* wild type (5-year OS = 49.2%, *p* = 0.003). In the multivariable analysis, *KRAS* mutation was an independent strong predictor of poor OS. *KRAS* mutation analysis should be included in the routine pathologic evaluation of resected hilar cholangiocarcinoma in order to better stratify prognosis.

**Abstract:**

*KRAS* mutation is reportedly associated with poor prognosis in patients with different cancer types. However, mutational data on hilar cholangiocarcinoma are few and controversial. The aim of this study was to evaluate the rate of *KRAS* mutations in a single-center homogeneous population resected for hilar cholangiocarcinoma and the subsequent impact on prognosis. *KRAS* mutation status was evaluated in 54 patients undergoing major hepatectomy combined with resection of the main biliary confluence and regional lymphadenectomy for hilar cholangiocarcinoma between 2001 and 2019. Among these 54 patients, 12 (22.2%) had a *KRAS* mutation. *KRAS* mutation was not related with pathologic characteristics of the tumor. Five-year overall survival (OS) in patients with *KRAS* mutation was significantly lower than that observed in patients with *KRAS* wild type (0 vs. 49.2%, respectively; *p* = 0.003). In the multivariable analysis; independent predictors of poor OS were *KRAS* mutation (HR = 5.384; *p* = 0.003) and lymph node metastases (HR = 2.805; *p* = 0.023). The results of our study suggested that *KRAS* mutation in hilar cholangiocarcinoma was not rarely observed. *KRAS* mutation was an independent strong predictor of poor OS. *KRAS* mutation analysis should be included in the routine pathologic evaluation of resected hilar cholangiocarcinoma in order to better stratify prognosis

## 1. Introduction

Perihilar cholangiocarcinoma (PHC) accounts for more than 50% of all cholangiocarcinomas [1,2]. It includes two separate subtypes: the hilar cholangiocarcinoma (Klatskin tumor), which arises from the extrahepatic main biliary confluence and the intrahepatic cholangiocarcinoma, with a liver mass invading the main biliary confluence [3]. Radical resection is the only treatment that can offer a chance of long-term survival, including main biliary confluence resection, associated with major hepatectomy and caudate lobe resection [4]. Resection with negative biliary margins (R0 resection) and presence of lymph node metastases represent the most significant independent prognostic factors [5,6].

In the current era of personalized medicine, molecular biomarkers have been evaluated as fundamental prognostic predictors that can define the type of chemotherapy and can select the best candidates for surgery. The *KRAS* oncogene is currently one of the most used molecular biomarkers in surgical oncology. Several studies have shown that *KRAS* mutation was documented in about 7–49% of patients with cholangiocarcinoma [7,8,9]. This rate may differ according to the anatomical location of the tumor: intrahepatic cholangiocarcinoma is generally associated with a significantly lower rate of *KRAS* mutation than that observed in patients with extrahepatic cholangiocarcinoma (7–22% vs. 37–46%, respectively) [7,8,9]. However, in most of these studies, the analysis of *KRAS* mutation status in patients with extrahepatic cholangiocarcinoma often included both PHC and distal cholangiocarcinoma, which are associated with different prognosis [7,8,9]. Moreover, it has been demonstrated that the rate of *KRAS* mutation in patients with PHC may vary according to the two subtypes. Indeed, *KRAS* mutation rate may be significantly higher in patients with hilar cholangiocarcinoma than that in patients with intrahepatic liver mass invading the hepatic hilum [10].

The significance of this study was to evaluate if *KRAS* mutation analysis may have a role in the routine pathologic evaluation of resected hilar cholangiocarcinoma in order to better stratify prognosis.

The aim of this study was to evaluate the rate of *KRAS* mutation in a single-center homogeneous population, resected for hilar cholangiocarcinoma and its impact on prognosis.

## 2. Materials and Methods

### 2.1. Inclusion Criteria

This is a retrospective observational single-center study. This study included patients who underwent major hepatectomy combined with resection of the main biliary confluence and regional lymphadenectomy for histologically proved PHC, at our unit, between January 2001 and December 2019. PHC includes two separate subtypes: the hilar cholangiocarcinoma (Klatskin tumor) which arises from the extrahepatic main biliary confluence and the intrahepatic cholangiocarcinoma with a liver mass invading the main biliary confluence. This study included only patients with the hilar cholangiocarcinoma subtype. Patients resected for intrahepatic cholangiocarcinoma involving the hepatic hilum were excluded.

Data were retrospectively extracted from a prospectively collected database established at our unit in January 1987 for all consecutive admissions related to possible liver resection. Inclusion criteria were: availability of *KRAS* mutation analysis performed at our University hospital; a minimum follow-up ≥3 years.

Between January 2001 and December 2019, 111 patients underwent major hepatectomy combined with resection of the main biliary confluence and regional lymphadenectomy for hilar cholangiocarcinoma at our unit. Of the resected patients, 7 (6.3%) died during the postoperative course and were excluded from the study. Among the remaining 104 patients, the *KRAS* mutation analysis was available in 54 patients, who are the subjects of our study.

Liver resections were defined according to the IHPBA terminology [11].

Preoperative biliary drainage was usually performed in jaundiced patients undergoing right or right extended hepatectomy. Preoperative biliary drainage for planned left-sided hepatectomies was selectively performed according to the age, general condition and comorbidities of the patient. When patients were referred to our unit without biliary drainage, our policy was to perform a unilateral biliary drainage of the future remnant liver by percutaneous approach [12].

Portal vein resection was not systematically performed according to the no-touch technique, described by Neuhaus et al. [13]. Portal vein resections were performed only when neoplastic portal invasion was intraoperatively confirmed and the portal vein could not be freed from the tumor during dissection of the hepatic pedicle.

All patients underwent regional lymphadenectomy, including hilar, pericholedochal, hepatic artery, periportal and superior retro-pancreatic lymph nodes.

The following data were collected for each patient: demographics; use and type of preoperative biliary drainage; use of preoperative portal vein embolization. Extent of bile duct involvement was defined by the Bismuth-Corlette classification [14]. Operative details included: type of resection; use of pedicle clamping; intraoperative blood transfusions. Early results included postoperative complications; late results included: 5-year overall survival (OS) rate.

### 2.2. Pathologic Data

Tumor staging was based on the TNM Classification by the UICC staging system, 8th Edition [15,16]. Pathologic data included presence of perineural invasion, radicality of resection (biliary margin status), invasion of biliary ducts of caudate lobe and lymph node involvement.

### 2.3. KRAS Mutation Analysis

As previously reported [17,18,19,20], *KRAS* mutation analysis was performed at the Anatomic Pathology Unit of our University Hospital. Tumoral tissue was identified in hematoxylin-and-eosin-stained sections of formalin-fixed, paraffin-embedded archival blocks. DNA was extracted from three 10 µm slides of paraffin-embedded tissue using the QIAamp DNA mini Kit (Qiagen, Milan, Italy). In order to minimize the contamination by normal cells, the tumor areas dissected for DNA and RNA extraction contained at least 70% of tumor cells. All KRAS mutations found were pathogenic mutations. As previously described [17,18,19,20], *KRAS* codons 12, 13, 59, 61, 117 and 146 were amplified in one PCR. Thermal cycling conditions were: 95 °C for 12 min followed by 40 cycles of 95 °C for 10 s, 55 °C for 20 s and 72 °C for 20 s. PCR conditions were as follows: primer concentration 200 nmol L^−1^, MgCl_2_ concentration 2 mmol L−1; 30 ng of genomic DNA and 12.5 µL of Eppendorf Prime mastermix (Eppendorf, Milan, Italy) in a final reaction volume of 25 µL. PCR products were electrophoresed in a 2.5% agarose gel, stained with ethidium bromide and visualized under UV light. Thereafter, 5 µL of PCR product was treated with ExoSAP-IT (GE Healthcare, Milan, Italy) following the manufacturer’s protocol, amplified with the BigDye Terminator version 3.1 cycle sequencing kit (Applied Biosystems, Milan, Italy) using the same primers of the amplification and sequenced with an ABI PRISM 3100-Avant Genetic Analyzer (Applied Biosystems) (Table 1).

All the analyzed samples were studied using the same technique which has a sensitivity of 5% (ability to identify mutations when the mutated DNA rate is 5% of the total DNA).

### 2.4. Adjuvant Chemotherapy

Adjuvant chemotherapy with gemcitabine was administered to patients with T3-T4 stage, with R1 resection or patients with lymph node metastases.

### 2.5. Primary Outcome

The primary outcome was the impact of *KRAS* mutation on overall survival following surgical resection for hilar cholangiocarcinoma.

### 2.6. Secondary Outcome

The secondary outcome was the incidence of *KRAS* mutation in resected patients with hilar cholangiocarcinoma.

### 2.7. Statistical Analysis

Continuous variables were reported as medians and ranges. Categorical variables were expressed in numbers and percentages. The OS was calculated from the date of liver resection until the date of death or censored at the last follow-up. Survival curves were generated using the Kaplan–Meier method and compared with the log-rank test. A multivariable regression analysis was performed to identify the independent prognostic factors for OS, using a Cox proportional hazards model with backward elimination for variables with *p* < 0.2 in univariate analysis. In all the analyses, a *p* < 0.05 was considered statistically significant. Analyses were carried out with SPSS 23.0 Software (SPSS Inc., Chicago, IL, USA).

## 3. Results

The characteristics observed in the study population are reported in Table 2. The mean age was 63 ± 11.7 years (range 33–80). Preoperative biliary drainage was performed in 32 patients (59.3%): by percutaneous approach in 22 patients (68.75%) and by endoscopic approach in 10 patients (31.25%). Right-sided hepatectomy was performed in 23 patients (42.6%) and left sided in 31 patients (57.4%) (Table 2). Preoperative right portal vein embolization was performed in 16 of the 23 right-sided hepatectomies (69.6%). Associated caudate lobe resection was performed in 39 patients (72.2%). Associated portal vein resection was performed in eight patients (14.8%).

### 3.1. Pathology

Tumor characteristics are shown in Table 3. R0 resection was performed in 39 patients (72.2%). Lymph node metastases were documented in 11 patients (20.4%). Neoplastic invasion of caudate lobe biliary ducts was found in 12 of the 39 associated caudate lobe resections (30.8%). Out of the eight performed portal vein resections, in four cases, a portal tumor invasion was confirmed at final pathology.

### 3.2. KRAS Mutation Analysis

Among the 54 resected patients, 12 (22.2%) had a *KRAS* mutation (Table 4). The most frequent mutations were found in codon 12 (14.8%).

### 3.3. Survival Analysis

**Figure 1:**
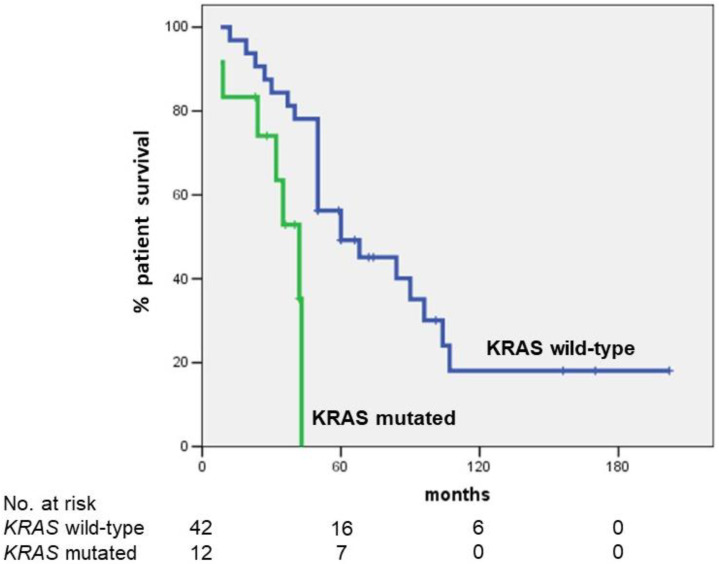
Five-year OS according to *KRAS* mutational status. Five-year OS in patients with *KRAS* mutation was significantly lower than that observed in patients with *KRAS* wild type (0 vs. 49.2%, respectively; *p* = 0.003). The median survival was 42 months in patients with *KRAS* mutation and 60 months in patients with *KRAS* wild type.

After a mean follow-up of 58.1 months, 16 patients were alive at the last follow-up. The 5-year OS for the total group of 54 patients was 42.0% (median OS: 50 months). Five-year OS in patients with *KRAS* mutation was significantly lower than that observed in patients with *KRAS* wild type (0 vs. 49.2%, respectively; *p* = 0.003) (Figure 1).

**Figure 2:**
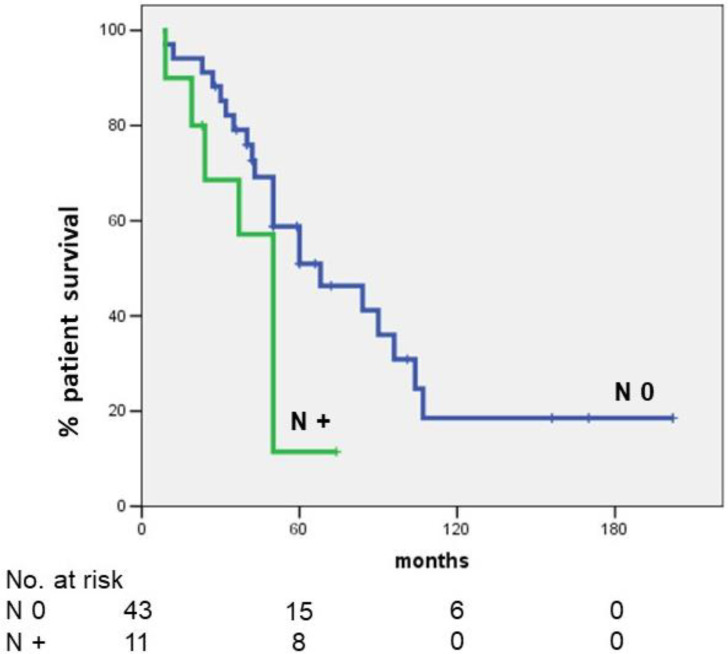
Five-year OS according to the presence of lymph node metastases. Five-year OS in patients with lymph node metastases was significantly lower than that observed in patients without lymph node metastases (11.4 vs. 51.0%, respectively; *p* = 0.023).

Five-year OS in patients with lymph node metastases was significantly lower than that observed in patients without lymph node metastases (11.4 vs. 51.0%, respectively; *p* = 0.023) (Figure 2).

Recurrence was documented in 33 patients (61.1%). Type of recurrence was available in 21 patients: peritoneal carcinomatosis (eight patients), local recurrence (five patients), lymph node metastases (three patients), pulmonary metastases (three patients), liver metastases (one patient) and seeding metastases (one patient). The 5-year recurrence-free survival was 37.5%.

In the multivariable analysis, independent predictors of poor OS were *KRAS* mutation (HR = 5.384; *p* = 0.003) and lymph node metastases (HR = 2.805; *p* = 0.023) (Table 5).

*KRAS* mutation status was not significantly different according to the stage of the tumor (Table 6).

## 4. Discussion

This single-center study showed that *KRAS* mutation was an independent predictor of poor OS following radical resection for hilar cholangiocarcinoma. Indeed, the 5-year OS for the total group of patients resected for PHC was 42.0%. The 5-year OS was significantly different in patients with *KRAS* wild type than in patients with *KRAS* mutation (49.2% vs. 0, respectively; *p* = 0.003). Moreover, in the multivariable analysis, *KRAS* mutation together with lymph node metastases were the strongest predictors of poor OS.

*KRAS* mutation is reportedly associated with poor prognosis in patients with different cancer types [21,22,23,24,25,26]. However, mutational data on PHC are few and controversial. The rate of *KRAS* mutation in patients with cholangiocarcinoma may range between 7% and 49% [7,8,9]. This wide range of discrepancy in the literature is due to different frequencies of mutation associated with different anatomical tumor locations. Indeed, it has been demonstrated that *KRAS* mutational frequency is different between intrahepatic cholangiocarcinoma and extrahepatic cholangiocarcinoma [27]. In a recent paper by Ruzzenente et al. [10], out of the 35 resected patients with intrahepatic cholangiocarcinoma, only 3 (8.6%) presented a *KRAS* mutation. On the other hand, the frequency of *KRAS* mutation in an international multicenter cohort of 189 patients with extrahepatic cholangiocarcinoma was 36.7% [7]. Due to this quite high frequency of mutation, *KRAS* mutation analysis in patients with extrahepatic cholangiocarcinoma may have a role in the stratification of prognosis. However, extrahepatic cholangiocarcinoma includes two different types of tumors that require different types of surgical resection: the PHC and the distal cholangiocarcinoma. Most of the published studies reported the *KRAS* mutation frequency of extrahepatic cholangiocarcinoma without differentiating between PHC and distal cholangiocarcinoma, with consequent controversial results and bias in prognosis [8,9]. In a recent paper by Zheng et al. [28], the authors analyzed the differences in *KRAS* mutational status between PHC and distal cholangiocarcinoma in a Chinese population, including 70 PHC and 108 distal tumors. In the entire population, *KRAS* mutation was the second-most-commonly detected mutation (32%) after TP53 (56%) [28]. In that study, *KRAS* mutation frequency was significantly higher in distal tumors than in PHC (<45% vs. <25%, respectively; *p* < 0.01). However, the reported *KRAS* mutation frequency in patients with PHC may also vary according to different studies. Sturm et al. reported a *KRAS* mutation frequency of 40.7% in patients with PHC [29]. The discrepancy of *KRAS* mutation frequency in PHC shows that simply differentiating PHC from distal cholangiocarcinoma may not be sufficient. Indeed, PHC also includes two different types of tumors: the hilar cholangiocarcinoma (Klatskin tumor), which arises from the extrahepatic main biliary confluence, and the intrahepatic cholangiocarcinoma, with a liver mass invading the main biliary confluence. These two subtypes of tumors are both included in the term perihilar cholangiocarcinoma, but they may show different frequencies in *KRAS* mutation. The first study that differentiated these two subtypes was the paper by Ruzzenente et al. [10]. This study showed that *KRAS* mutation was observed in 47.4% of the 38 resected patients with hilar cholangiocarcinoma and in 22.2% of the 18 resected patients with intrahepatic cholangiocarcinoma invading the hilum.

Our study evaluated the *KRAS* mutation frequency in a homogeneous single-center population resected for hilar cholangiocarcinoma. All these patients were resected at the same unit and *KRAS* mutation analysis was performed with the same technique by the same Anatomic Pathology Unit. Moreover, the study analyzed the frequency of *KRAS* mutation and its impact on OS in patients resected for a specific subtype of PHC: the hilar cholangiocarcinoma. Out of the 54 resected patients, 12 (22.2%) had a *KRAS* mutation. The presence of mutation was a strong independent predictor of poor OS. Indeed, patients with *KRAS* mutation presented a null 5-year OS that was significantly lower than that observed in patients with *KRAS* wild type (5-year OS = 49.2%). In this series, all patients underwent regional lymphadenectomy with a mean number of harvested lymph nodes of 5.7. Moreover, the rate of R0 resection was 72.2%. This means that all patients underwent a correct radical resection with an appropriate number of harvested lymph nodes [6], associated with correct staging. Our study confirmed that lymph node metastases were a strong independent predictor of poor OS: 5-year OS in patients with lymph node metastases was significantly lower than that observed in N0 patients (11.4% vs. 51.0%, respectively; *p* = 0.039).

Interestingly, in our population, the presence of *KRAS* mutation was not related with pathologic characteristics of the tumor: the T stage, presence of lymph node metastases, presence of perineural invasion and caudate lobe invasion. This means that *KRAS* mutation analysis should be included in the prognostic stratification of patients resected for PHC, in order to select patients for adjuvant chemotherapy in case of good pathologic results, such as T1–T2 stage or absence of lymph node metastases. Moreover, *KRAS* mutation was associated with a higher rate of systemic recurrence than that observed in patients with *KRAS* wild type, without reaching a statistically significant difference due to the small sample size (75.0% vs. 58.8%, respectively; *p* = 0.548). However, this result may confirm that *KRAS* mutation is associated with an aggressive behavior of hilar cholangiocarcinoma, especially the development of systemic recurrence, with consequent significantly lower OS.

The present study has some limitations. It is a retrospective study, which collected a relatively small number of patients. However, by analyzing the literature, it is evident that large cohorts of patients come from multicenter studies with different methods of *KRAS* analysis. These studies usually collect data from extrahepatic cholangiocarcinomas without differentiating PHC from distal tumors and they may be associated with bias in prognostic results.

## 5. Conclusions

Our study showed that *KRAS* mutation in hilar cholangiocarcinoma was not rarely observed. The frequency of mutation in our series was 22.2% and it was not related with pathologic characteristics of the tumor. *KRAS* mutation was an independent strong predictor of poor OS. *KRAS* mutation analysis should be included in the routine pathologic evaluation of resected hilar cholangiocarcinoma in order to better stratify prognosis.

## Figures and Tables

**Table 1:** *KRAS* primers of amplification: k2, couples A50–A51; 646–647.

Primer	Sequence
A50	TGTTCTAATATAGTCACATTTTCATT
A51	TCCTGCACCAGTAATATGC
646	GCCTGCTGAAAATGACTGAAT
647	TTATCTGTATCAAAGAATGGTC

**Table 2:** Characteristics of the 54 resected patients for PHC.

Variable	No. (%)/Median [Range]
Age	65 [33–80]
Gender; (men/women)	32/22
Extent of biliary involvement	
(Bismuth-Corlette classification)	
Type 1	1 (1.8)
Type 2	4 (7.4)
Type 3	48 (89.0)
Type 3a	24 (50.0)
Type 3b	24 (50.0)
Type 4	1 (1.8)
Preoperative biliary drainage	
Yes	32 (59.3)
No	22 (40.7)
Percutaneous approach	22 (68.75)
Endoscopic approach	10 (31.25)
Preoperative right portal vein embolization	16/23 right-sided hepatectomies (69.6)
Type of liver resection	
Right-sided hepatectomy	23 (42.6)
Right hepatectomy	7
Right hepatectomy with S4	2
Right hepatectomy with S1	7
Right hepatectomy with S4-1	7
Left-sided hepatectomy	31 (57.4)
Left hepatectomy	6
Left hepatectomy with S1	25
Associated caudate lobe resection	39 (72.2)
Pedicle clamping	30 (55.5)
Intraoperative blood transfusions	10 (18.5)
Postoperative complications	22 (40.7)
Adjuvant chemotherapy	20 (37.0)

**Table 3:** Pathological characteristics of the 54 resected patients for PHC.

Variable	No. (%)/Mean ± SD [Range]
Margin status	
R0	39 (72.2)
R1	15 (27.8)
Perineural invasion	37 (68.5)
Caudate lobe invasion	12/39 caudate lobe resection (30.8)
T stage, 8th edition	
T1	3 (5.6)
T2a	8 (14.8)
T2b	35 (64.8)
T3	4 (7.4)
T4	4 (7.4)
Harvested lymph node	5.7 ± 4.9 [1–20]
Lymph node status	
Negative	43 (79.6)
N1	10 (18.5)
N2	1 (1.9)
Metastatic lymph nodes (among the total number of documented positive lymph nodes)	2.1 ± 1.5 [1–6]

**Table 4:** *KRAS* mutation analysis.

*KRAS* Mutation	No. (%)
Codon 12	8 (14.8)
p.G12D	5 (9.3)
p.G12V	3 (5.5)
Codon 13	2 (3.7)
p.G13D	2 (3.7)
p.(Gln61Xaa)	2 (3.7)

**Table 5:** Univariate and multivariable analysis of OS in 54 resected patients for PHC.

			Univariate Analysis	Multivariable Analysis	
Variable	No. (%)	5-Year OS (%)	*p*-Value	HR (95% CI)	*p*-Value
Age (yr)			0.923		
<70	36 (66.7)	43.4			
≥70	18 (33.3)	39.9			
Gender			0.735		
Male	32 (59.3)	40.9			
Female	22 (40.7)	41.3			
Bismuth type			0.994		
1–2	5 (9.3)	33.3			
3–4	49 (90.7)	42.4			
Preoperative biliary drainage			0.366		
Yes	32 (59.3)	43.0			
No	22 (40.7)	52.5			
Preoperative right portal vein embolization			0.793		
Yes	16 (29.6)	43.8			
No	38 (70.4)	41.3			
Type of liver resection			0.619		
Right-sided resection	23 (42.6)	50.5			
Left-sided resection	31 (57.4)	36.4			
Associated caudate lobe resection			0.416		
Yes	39 (72.2)	45.4			
No	15 (27.8)	31.8			
Portal vein resection			0.394		
Yes	8 (14.8)	41.7			
No	46 (85.2)	41.8			
Pedicle clamping			0.642		
Yes	30 (55.5)	41.8			
No	24 (44.5)	42.1			
Intraoperative blood transfusions			0.547		
Yes	10 (18.5)	37.0			
No	44 (81.5)	42.3			
Postoperative complications			0.760		
Yes	22 (40.7)	43.3			
No	32 (59.3)	41.2			
Margin status			0.464		
R0	39 (72.2)	44.6			
R1	15 (27.8)	33.3			
Perineural invasion			0.072		
Yes	37 (68.5)	49.4			
No	17 (31.5)	25.4			
Caudate lobe invasion			0.544		
Yes	12 (30.8)	59.3			
No	27 (69.2)	39.9			
T stage			0.591		
T1-T2	46 (85.2)	41.1			
T3-T4	8 (14.8)	50.0			
Lymph node status			0.039	2.805 (1.155–6.810)	0.023
Negative	43 (79.6)	51.0			
Metastatic	11 (20.4)	11.4			
Adjuvant chemotherapy			0.269		
Yes	20 (37.0)	50.5			
No	34 (63.0)	37.0			
*KRAS* mutation status			0.003	5.384 (1.755–16.519)	0.003
wild-type	42 (77.8)	49.2			
mutated	12 (22.2)	0			
Time period			0.097		
2001–2010	25 (46.3)	46.8			
2011–2019	29 (53.7)	40.3			
Recurrence			0.011		
Yes	33 (61.1)	30.8			
No	21 (38.9)	72.7			

**Table 6:** Association between *KRAS* mutation status and patients’ characteristics and tumor stage.

Variable, No. (%)	*KRAS* Mutated (No. 12)	*KRAS* Wild-Type (No. 42)	*p*-Value
Age ≥ 70	4/12 (33.3)	14/42 (33.3)	1
Male	9/12 (75.0)	23/42 (55.0)	0.208
Preoperative biliary drainage	6/12 (50.0)	26/42 (61.9)	0.459
Right-sided resection	7/12 (58.3)	16/42 (38.1)	0.211
R0 resection	10/12 (83.3)	29/42 (69.0)	0.329
T stage (T3–T4)	1/12 (8.3)	7/42 (16.7)	0.473
Lymph node metastases	3/12 (25.0)	8/42 (19.0)	0.651
Presence of perineural invasion	8/12 (66.7)	29/42 (69.0)	0.875
Caudate lobe invasion	4/9 (44.4)	8/30 (26.7)	0.310
Bismuth type 3–4	12/12 (100)	37/42 (88.1)	0.209
Type of recurrence (data available on 21 pts.)			0.548
Local recurrence	1/4 (25.0)	7/17 (41.2)	
Systemic recurrence	3/4 (75.0)	10/17 (58.8)	

## Data Availability

Not applicable.
